# *me31B* regulates stem cell homeostasis by preventing excess dedifferentiation in the *Drosophila* male germline

**DOI:** 10.1242/jcs.258757

**Published:** 2021-07-22

**Authors:** Lindy Jensen, Zsolt G. Venkei, George J. Watase, Bitarka Bisai, Scott Pletcher, Cheng-Yu Lee, Yukiko M. Yamashita

**Affiliations:** 1 Life Sciences Institute, Department of Molecular and Integrative Physiology, University of Michigan Ann Arbor, MI 48109, USA; 2 Whitehead Institute for Biomedical Research, Massachusetts Institute of Technology, Department of Biology, Cambridge, MA 02142, USA; 3 Howard Hughes Medical Institute, Cambridge, MA 02142, USA

**Keywords:** Dedifferentiation, Spermatogenesis, *Drosophila*, Germ cells

## Abstract

Tissue-specific stem cells maintain tissue homeostasis by providing a continuous supply of differentiated cells throughout the life of organisms. Differentiated/differentiating cells can revert back to a stem cell identity via dedifferentiation to help maintain the stem cell pool beyond the lifetime of individual stem cells. Although dedifferentiation is important for maintaining the stem cell population, it is speculated that it underlies tumorigenesis. Therefore, this process must be tightly controlled. Here, we show that a translational regulator, *me31B*, plays a critical role in preventing excess dedifferentiation in the *Drosophila* male germline: in the absence of *me31B*, spermatogonia dedifferentiate into germline stem cells (GSCs) at a dramatically elevated frequency. Our results show that the excess dedifferentiation is likely due to misregulation of *nos*, a key regulator of germ cell identity and GSC maintenance. Taken together, our data reveal negative regulation of dedifferentiation to balance stem cell maintenance with differentiation.

## Abstract (precis)

**Summary:** *me31B* is identified as a negative regulator of dedifferentiation from spermatogonia to germline stem cells in the *Drosophila* testis via its ability to downregulate *nos* expression.

## INTRODUCTION

Tissue-specific adult stem cells play a critical role in sustaining tissue homeostasis by continuously providing differentiated cells throughout the life of organisms (He et al., 2009; Nystul and Spradling, 2006). The loss of stem cells or their functions underlie tissue degeneration under physiological and pathological conditions. The stem cell pool is primarily maintained by self-renewal. In addition, dedifferentiation, a process whereby differentiated and/or differentiating cells revert back to a stem cell identity, also helps to maintain the stem cell population beyond the lifetime of individual stem cells (de Sousa and de Sauvage, 2019; Merrell and Stanger, 2016). However, the misregulation of dedifferentiation is thought to underlie tumorigenesis (Landsberg et al., 2012; Schwitalla et al., 2013). Therefore, dedifferentiation must be tightly controlled to ensure stem cell maintenance, while preventing transformation. However, the molecular mechanisms that regulate dedifferentiation are not well understood.

**Fig. 1.**
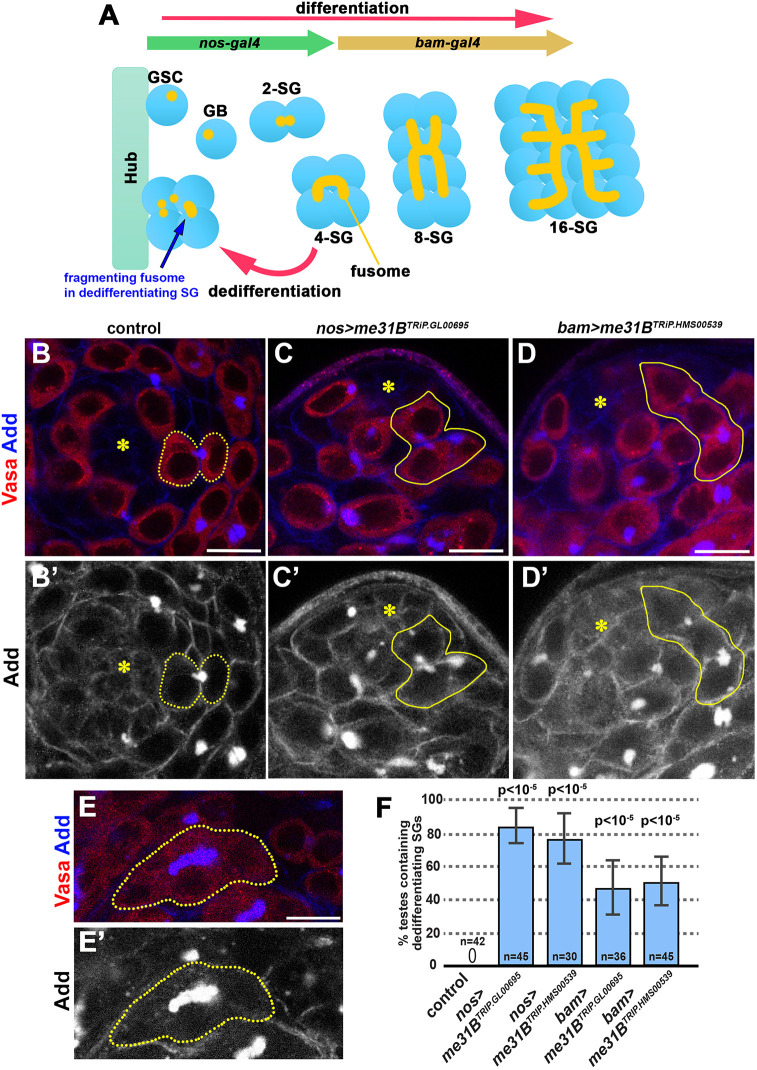
**Me31B knockdown leads to excessive dedifferentiation in the *Drosophila* testis.** (A) *Drosophila* spermatogenesis. GSCs are attached to the hub cells, which provide signaling ligands required for GSC self-renewal. Asymmetric GSC division generates a GSC and GBs that undergo four rounds of mitotic divisions to create two-, four-, eight- and 16-cell SGs. The 16-cell SGs then proceed to spermatocyte stage, then to meiosis to produce sperm (not depicted). SGs can revert back to the GSC identity via dedifferentiation. During dedifferentiation, a cytoplasmic organelle called the fusome, which is normally a continuous structure that connects SGs, breaks apart. The fragmenting fusome in the dedifferentiating SG is indicated by a blue arrow. The *nos-gal4* driver is expressed in GSCs until the four-cell SGs, whereas *bam-gal4* is expressed after the four-cell SG stage. RNAi initiated by *nos-gal4* typically endures after *nos-gal4* expression ceases, due to persistence of RNAi (Bosch et al., 2016). (B-D′). Apical tip of the testis stained for Vasa (red, germ cells) and Adducin-like (Add, blue, fusome) in controls (B,B′) and *nos>me31B^TRiP.GL00695^* (C,C′) and *bam>me31B^TRiP.HMS00539^* (D,D′) knockdown lines. Both RNAi lines were similarly effective, and experiments were conducted using both RNAi lines (unless the genetics crosses were too complicated to generate a desired genotype). Throughout the paper, examples may be shown only with one RNAi construct, but the results were confirmed by using both constructs unless otherwise noted. Yellow dotted lines indicate GSC-GB pair (B), and yellow solid lines indicate dedifferentiating SG cyst (C,D). Fusomes are fragmented in dedifferentiating SG cysts (C,D). The hub is indicated by the asterisks. (E) An example of a continuous fusome observed in differentiating SGs (a four-cell cyst). (F) Frequency of testes (%) containing dedifferentiating SG cysts attached to the hub with ≥3 germ cells and fragmented fusomes in control versus *me31B* knockdown testes. *n*=number of testes scored. Data are mean±s.d. *P*-value from Fisher's exact test is provided compared to control. Scale bars: 10 µm.

The *Drosophila* testis serves as an excellent model system to study dedifferentiation. Notably, this model offers unambiguous identification of stem cells [germline stem cells (GSCs)] and their differentiating progeny (Fuller and Spradling, 2007; Yamashita, 2018). GSCs are attached to postmitotic somatic hub cells, which function as a major component of the stem cell niche (Fig. 1A). The hub cells secrete two major signaling ligands that promote GSC self-renewal: a cytokine-like ligand Upd that activates the JAK-STAT (Janus kinase-signal transducer and activator of transcription) pathway, and a BMP (bone morphogenic protein) ligand Dpp that activates the downstream Tkv receptor (Kawase et al., 2004; Kiger et al., 2001; Schulz et al., 2004; Shivdasani and Ingham, 2003; Tulina and Matunis, 2001). Upon GSC divisions, daughter cells that are displaced away from the hub initiate differentiation as gonialblasts (GBs), which then continue with proliferative mitotic divisions (or transit-amplifying divisions) as spermatogonia (SGs) before entering a meiotic program as spermatocytes (SCs). SG divisions are characterized by incomplete cytokinesis, connecting all sister cells as a cluster (i.e. cyst). A membranous organelle called the fusome runs through the stabilized contractile ring, called a ring canal, connecting SGs within a cyst (Fig. 1A; Yamashita, 2018).

Although GSCs are maintained relatively stably through consistent asymmetric divisions, which generate one GSC and one GB (Yamashita et al., 2003), GSCs can occasionally be lost (Wallenfang et al., 2006). Upon GSC loss, SGs can respond to niche vacancy, and dedifferentiate to replenish the GSC pool. During dedifferentiation of SGs, the fusome that connects SGs fragments into a more spherical structure, referred to as ‘spectrosome’, as typically observed in GSCs (Fig. 1A) (Brawley and Matunis, 2004). Fragmenting fusomes in >2 cell SGs are observed only during dedifferentiation, not during differentiation, and these features can be used to unambiguously identify dedifferentiating SGs without lineage tracing (Brawley and Matunis, 2004; Sheng et al., 2009; Sheng and Matunis, 2011). Dedifferentiation was first shown in an experiment that artificially removed all GSCs via transient overexpression of Bam, a master regulator of differentiation (Brawley and Matunis, 2004; Sheng et al., 2009; Sheng and Matunis, 2011). Although temporally controlled overexpression of Bam induced all GSCs to differentiate, withdrawal of Bam allowed SGs to repopulate the stem cell niche and produce GSCs. Subsequently, it was shown that SG dedifferentiation occurs naturally and increases during aging in unperturbed tissues (Cheng et al., 2008), suggesting that dedifferentiation is likely a mechanism that helps to maintain the GSC population throughout the lifetime of organisms, particularly with age. More recent work showed that dedifferentiation is important for sustaining the GSC population under conditions that repeatedly induce GSC replenishment and challenge tissue homeostasis, such as cycles of starvation and refeeding (Herrera and Bach, 2018). SG dedifferentiation under these conditions required Jun N-terminal kinase (JNK) signaling (Herrera and Bach, 2018). However, whether mechanisms exist to prevent excess dedifferentiation remain poorly understood.

*Maternal expression at 31B* (*me31B*) encodes an RNA helicase of the DEAD-box family that regulates translation (Kugler et al., 2009; Kugler and Lasko, 2009; Nakamura et al., 2001). In particular, *me31B* silences the translation of oocyte-localizing mRNAs, such as *oskar*, in nurse cells prior to their transport to the oocyte (McDermott et al., 2012; Nakamura et al., 2001). *me31B* has also been shown to repress translation of *nanos* (*nos*) (Gotze et al., 2017; Jeske et al., 2011), a translational regulator that is critical for germ cell specification and maintenance of GSCs (Li et al., 2009; Wang and Lin, 2004). Here, we show that *me31B* is a critical negative regulator of dedifferentiation in the *Drosophila* testis. In the absence of *me31B*, SGs frequently dedifferentiated even in the absence of known triggers, such as the induced removal of GSCs. We further show that *me31B* suppresses SG dedifferentiation by repressing *nos*. Our study reveals that dedifferentiation is actively repressed under normal conditions, likely to protect the native GSC population, and identifies *me31B* as a previously unknown negative regulator of dedifferentiation.

## RESULTS

### *me31B* prevents excess dedifferentiation of SG in *Drosophila* testes

To study the role of *me31B* in the testis, we used two independent RNAi constructs [*UAS-me31B^TRiP.GL00695^* and *UAS-me31B^TRiP.HMS00539^*, available from Bloomington *Drosophila* Stock Center (see Materials and Methods)]. Using these constructs and the *nos-gal4* driver, we knocked down *me31B* in germ cells (Fig. S1, *nos-gal4*>*UAS-me31B^TRiP.GL00695^* and *nos-gal4>UAS-me31B^TRiP.HMS00539^*, hereafter *nos>me31B^TRiP.GL00695^* and *nos>me31B^TRiP.HMS00539^*, respectively, or simply *nos>me31B^RNAi^*, as essentially the same results were obtained with both RNAi constructs). We found that Me31B-GFP was expressed in both germline and somatic cells in the testis, and the GFP signal was substantially reduced in the germline upon expression of the RNAi construct using *nos-gal4*, confirming the efficiency of these RNAi constructs (Fig. S1). Although Me31B has been reported to be a component of nuage (germ granules) (DeHaan et al., 2017; Liu et al., 2011; Thomson et al., 2008), we observed diffuse cytoplasmic localization of Me31B-GFP in germ cells in the adult testis and Me31B-GFP did not colocalize with the nuage marker Vasa in control flies. Moreover, *me31B* knockdown did not affect nuage morphology (Fig. S1).

As expected, GSCs in control testes surrounded the hub and were either single cells or connected to their immediate daughter cells (GBs) prior to completion of cytokinesis (Fig. 1B). Intriguingly, *nos>me31B^RNAi^* testes often contained dedifferentiating SG cysts that were attached to the hub cells (Fig. 1B). Their identity as dedifferentiating SG cysts is based on the fact that they contained ≥3 germ cells that were connected to each other (Fig. 1C-D′; see Materials and Methods for details for identifying dedifferentiating cysts). The fusomes in these SG cysts at the hub in *nos>me31B^RNAi^* testes were fragmented (Fig. 1C-D′), a well-established hallmark of dedifferentiating SGs (Brawley and Matunis, 2004; Sheng et al., 2009; Sheng and Matunis, 2011), rather than continuous as in differentiating SGs (Fig. 1E,E′). We observed dedifferentiating SG cysts, identified by their fragmented fusomes and attachment to the hub, in ∼80% of *nos>me31B^RNAi^* testes but not in any control testes (Fig. 1F). The number of SGs within dedifferentiating SG cysts was not always 2^n^: often they contained three SGs, indicating that some SGs might have already dedifferentiated into single GSCs or died during dedifferentiation.

We considered two possibilities that could explain this phenotype. First, *me31B* may be required in SGs to directly prevent their dedifferentiation. Second, *me31B* may be required to maintain GSCs in the niche, which would indirectly prevent SG dedifferentiation. To determine whether *me31B* acts directly in SGs, we used the *bam-gal4* driver to deplete *me31B* only in the four-cell SGs and later stages (Chen and McKearin, 2003b). We found that ∼50% of *bam>me31B^RNAi^* testes contained dedifferentiating SG cysts (Fig. 1D,F). These results demonstrate that *me31B* is required in SGs in a cell-autonomous manner to prevent their dedifferentiation; however, we note that the frequency of dedifferentiation is higher when RNAi constructs were driven by *nos-gal4* than by *bam-gal4*, suggesting that *me31B* may have additional functions in early germ cells to indirectly prevent dedifferentiation (see below).

### Dedifferentiating SGs activate BMP signaling

GSC identity in the *Drosophila* testis is specified by JAK-STAT and BMP signaling (Kawase et al., 2004; Kiger et al., 2001; Schulz et al., 2004; Shivdasani and Ingham, 2003; Tulina and Matunis, 2001). We examined whether the activation of these pathways was altered upon knockdown of *me31B*.

**Fig. 2.**
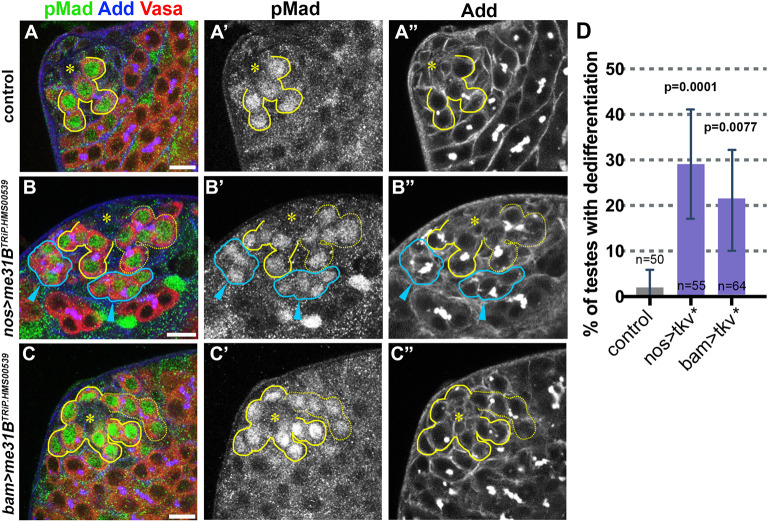
**BMP signaling is upregulated upon knockdown of *me31B.*** (A-C″) Apical tip of the testes in control (A), *nos-gal4>me31B^TRiP.HMS00539^* (B) or *bam-gal4> me31B^TRiP.HMS00539^* (C) stained for pMad (green), Vasa (red) and Adducin-like (blue). Scale bars: 10 µm. The hub is indicated by the asterisks. GSCs and connected GBs are indicated by yellow lines. Dedifferentiating cysts that are attached to the hub are indicated by yellow dotted lines. Dedifferentiating cysts that are not yet attached to the hub are indicated by blue lines and arrowheads. (D) Ectopic expression of constitutive active Tkv (Tkv*) either by *nos-gal4* driver or *bam-gal4* driver results in elevated dedifferentiation. *n*=number of testes scored. Data are mean±s.d. *P*-value from Fisher's exact test is provided compared to control.

In wild-type testes, activation of BMP signaling triggers phosphorylation of Mad (pMad) in GSCs and in GBs that are still connected to GSCs (Kawase et al., 2004) (Fig. 2A-A″). We found that knockdown of *me31B*, either by *nos-gal4* or *bam-gal4*, resulted in a high pMad signal in germ cells outside GSCs and GBs (Fig. 2B-C″). Moreover, in *me31B^RNAi^* testes, we observed high pMad signal in all the germ cells within a dedifferentiating SG cyst attached to the hub (Fig. 2B-C″) and even in SGs that were not yet attached to the hub (Fig. 2B-B″). We observed pMad^+^ germ cells outside the niche in only 7.7% of control testis (*n*=39 testes), but in over 50% of *me31B^RNAi^* testes (91.7% in *nos*>*me31B^TRiP.HMS00539^*, *n*=48; 66.7% in *nos*>*me31B^TRiP.GL00695^*, *n*=18; 58.8% in *bam*>*me31B^TRiP.HMS00539^*, *n*=34; 54.8% in *bam*>*me31B^TRiP.GL00695^*, *n*=31). These results indicate that the activation of BMP signaling precedes the re-acquisition of GSC identity during dedifferentiation in *me31B^RNAi^* testes, and may mediate dedifferentiation. Indeed, we found that overexpression of constitutively active Tkv (Tkv*) (Nellen et al., 1996), the receptor of BMP ligands, either by *nos-gal4* or *bam-gal4*, was sufficient to induce dedifferentiation (Fig. 2D). Taken together, we propose that *me31B* may prevent dedifferentiation of SGs by directly or indirectly downregulating BMP signaling.

In contrast to the deregulation of BMP signaling upon knockdown of *me31B*, we found that GSCs in *bam>me31B^RNAi^* testes had similar STAT expression as control testes (Fig. S2A-B″), suggesting that dedifferentiation induced in *bam>me31B^RNAi^* testes is not due to altered STAT signaling. However, STAT expression was reduced in GSCs of the *nos>me31B^RNAi^* testes compared to control (Fig. S2C-D″), suggesting that *me31B* may have an additional role in GSCs to maintain STAT activation (see Discussion).

### Knockdown of *me31B* leads to misregulation of *nos* expression

Previous work showed that Me31B silences *nos* mRNA translation during embryonic development of *Drosophila* (Gotze et al., 2017; Jeske et al., 2011). In the adult germline, Nos instructs germ cell identity and GSC maintenance via translational repression of critical targets, such as Bam (Li et al., 2009; Wang and Lin, 2004), and a regulatory feedback exists between *nos*, Mad and *bam* to control germ cell differentiation (Harris et al., 2011).

**Fig. 3.**
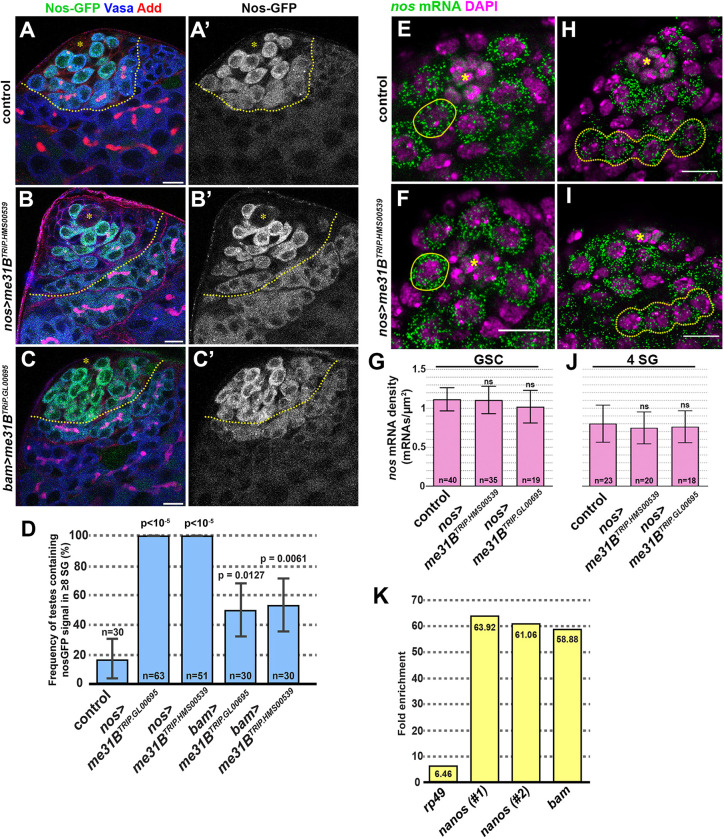
**Me31B binds to *nos* and *bam* mRNA to promote SG differentiation.** (A-C′) Apical tip of the testes expressing *nos-GFP* under the control of endogenous promoter and 3′ UTR, stained for Vasa (blue) and Adducin-like (red). Control (A,A′), *nos>me31B^TRiP.HMS00539^* (B,B′) or *bam>me31B^TRiP.GL00695^* (C,C′). The hub is indicated by the asterisks. The boundary between four-cell and eight-cell SGs is indicated by yellow dotted lines. (D) The frequency of the testes that contains Nos-GFP^+^ ≥8-cell SGs. *n*=number of testes scored. *P*-value from Fisher's exact test is provided compared to control. (E,F) Apical tip of the testes from control (E) or *nos>me31B^TRiP.HMS00539^* (F) probed for *nos* mRNA with single molecule RNA *in situ* hybridization with representative GSCs encircled. *nos* mRNA (green), DNA (magenta). The hub is indicated by the asterisks. (G) The quantification of *nos* mRNA signals in GSCs from control and *nos>me31B^RNAi^* (*nos* mRNA molecules µm^−2^ at the central cross-section). The indicated number of GSCs have been quantified from 6-9 testes of biological duplicates for each genotype. *P* values of Welch's unequal variances *t*-tests (unpaired, two-tailed) are indicated. (H,I) Apical tip of the testes from control (H) or *nos>me31B^TRiP.HMS00539^* (I) probed for *nos* mRNA with single molecule RNA *in situ* hybridization with representative four-cell SGs encircled. *nos* mRNA (green), DNA (magenta). The hub is indicated by the asterisks. (J) The quantification of *nos* mRNA signals in four-cell SGs from control and *nos>me31B^RNAi^* (*nos* mRNA molecules µm^−2^ at the central cross-section). The indicated number of GSCs have been quantified from five testes of biological duplicates for each genotype. *P* values of Welch's unequal variances *t*-tests (unpaired, two-tailed) are indicated. (K) Me31B-GFP RIP-qPCR probed for two sets of primers for *nos* mRNA and a primer set for *bam* mRNA, demonstrating that both *nos* mRNA and *bam* mRNA are highly enriched upon pulldown of Me31B-GFP protein. s.d for each primer set is as follows: *RP49*, 0.049237; *nos* #1, 0.046968; *nos* #2, 0.026151; *bam*, 0.065409. Data are mean±s.d. Scale bars: 10 µm. ns, not significant.

To investigate whether Me31B might regulate *nos* mRNA translation during spermatogenesis, we examined Nos protein levels upon knockdown of *me31B*. In control testes, we detected Nos protein in early-stage germ cells (GSC to four-cell stage SGs) (Fig. 3A). In contrast, upon knockdown of *me31B* either by *nos-gal4* or *bam-gal4*, we observed Nos protein even in eight-cell SGs (Fig. 3B-D), consistent with Me31B downregulating *nos* mRNA translation in the *Drosophila* testis. Nos and Bam, a master regulator of differentiation (McKearin and Ohlstein, 1995; McKearin and Spradling, 1990), are expressed in a reciprocal manner and act antagonistically in stem cell maintenance and differentiation in the *Drosophila* germline (Chen and McKearin, 2005; Li et al., 2009). Indeed, *me31B* knockdown in the testes led to delayed Bam expression and a dramatic increase in the frequency of four-cell SGs that lacked Bam protein (Fig. S3).

To determine whether Me31B regulates *nos* mRNA levels, we conducted single molecule RNA *in situ* hybridization to quantify *nos* mRNA levels (see Materials and Methods). We did not detect any difference in *nos* mRNA levels when comparing control versus *nos*>*me31B^RNAi^* testes, either in GSCs or SGs (Fig. 3E-J), suggesting that Me31B does not regulate *nos* mRNA levels. Taken together, these results suggest that Me31B regulates *nos* mRNA translation but not mRNA levels, consistent with other contexts in which Me31B acts as a regulator of translation (Nakamura et al., 2001; Peter et al., 2019; Wang et al., 2017).

To determine whether Me31B might regulate *nos* mRNA translation via direct binding, we performed RNA immunoprecipitation (RIP)-qPCR with testes expressing Me31B-GFP or GFP as a control [see Materials and Methods: note that we also ectopically expressed Dpp to cause SG overproliferation (Kawase et al., 2004; Schulz et al., 2004; Shivdasani and Ingham, 2003) to increase the starting material]. We found that *nos* mRNA co-immunoprecipitated with Me31B-GFP (Fig. 3K). Interestingly, *bam* mRNA also co-immunoprecipitated with Me31B-GFP (Fig. 3K). These results indicate that *nos* mRNA is likely a direct target of Me31B in the testis, and identify *bam* mRNA as a potential additional target. Overall, we conclude that *me31B* prevents dedifferentiation of SGs by reducing Nos protein levels and potentially increasing Bam protein levels.

### *nos* is necessary and sufficient for dedifferentiation

**Fig. 4.**
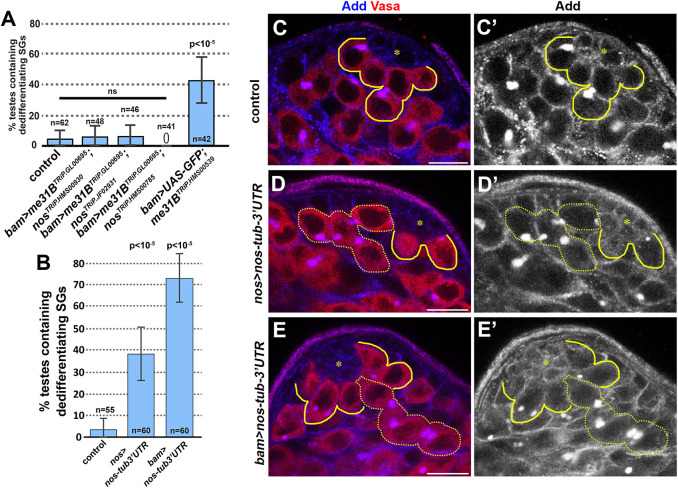
***nos* is necessary and sufficient for dedifferentiation.** (A) Frequency of testes containing dedifferentiating cysts in the indicated genotypes. Knockdown of *nos* diminishes dedifferentiation due to *me31B* knockdown. *n*=number of testes scored. (B) Frequency of testes containing dedifferentiating cysts upon ectopic expression of *nos* with *tubulin 3′UTR* (*nos-tub3′UTR*) driven by *nos-gal4* or *bam-gal4*. *P*-value from Fisher's exact test is provided compared to control. (C-E′) Apical tip of testes from control testis (C,C′), testis expressing *nos-tub3′UTR* by *nos-gal4* (D,D′) or *bam-gal4* (E,E′). GSCs and connected GBs are indicated by solid yellow lines, and dedifferentiating cysts are indicated by dotted yellow lines. The hub is indicated by the asterisks. Data are mean±s.d. Scale bars: 10 µm. ns, not significant.

Based on the results described above, we hypothesized that Me31B prevents dedifferentiation in late SGs by silencing *nos* mRNA translation. This hypothesis predicts that *nos* downregulation would rescue the elevated dedifferentiation caused by knockdown of *me31B*. Indeed, we found that simultaneous knockdown of *nos* and *me31B* greatly reduced dedifferentiation to the level of the control (Fig. 4A; Fig. S4). These data suggest that *nos* is the major functional target of *me31B* in preventing dedifferentiation. To verify that the reduced dedifferentiation in the double knockdown lines is not due to the presence of two UAS-driven transgenes and thus dilution of the gal4 driver, we tested a control genotype expressing *me31B^RNAi^* and a GFP transgene under the control of UAS. This genotype maintained the high frequency of dedifferentiation (Fig. 4A; Fig. S4). These results support the notion that *nos* is necessary for the dedifferentiation induced by depletion of *me31B*.

Moreover, we found that upregulation of *nos* was sufficient to induce dedifferentiation. We employed a *nos* transgene in which the 3′ untranslated region (UTR) is replaced by the *tubulin* 3′ UTR (*UAS-nos-tub3′UTR*), which disrupts the regulation of *nos* by translational repressors, such as Me31B (Gavis and Lehmann, 1994). When the *UAS-nos-tub3′UTR* transgene was expressed with the *nos-gal4* driver, we found that ∼40% of testes contained dedifferentiating SGs, as opposed to ∼3% in control (Fig. 4B-D′). Moreover, when the *UAS-nos-tub3′UTR* transgene was driven by *bam-gal4*, we observed an even higher frequency of dedifferentiation (∼70%) (Fig. 4B,E,E′). These results suggest that upregulation of *nos* is sufficient to induce dedifferentiation.

**Fig. 5.**
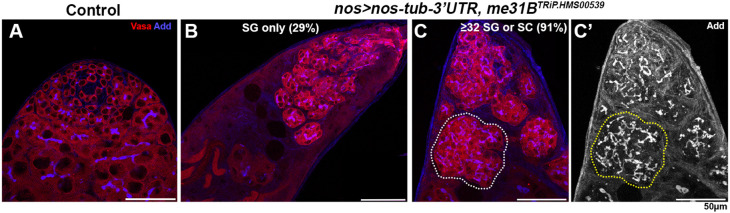
**Combination of *nos* upregulation and *me31B* knockdown blocks differentiation.** (A) Apical tip of the testes stained for Vasa (red) and Adducin-like (blue) in control (A) or *nos>nos-tub3′UTR, me31B^TRiP.HMS00539^* (B-C′). A cyst that contains ≥32 SGs is indicated by dotted lines in C and C′.

Interestingly, when *me31B* knockdown was combined with *nos-tub3′UTR* expression under the control of the *nos-gal4* driver, it led to a near complete block of differentiation (*nos>nos-tub3′UTR, me31B^TRiP.HMS00539^*; Fig. 5). The differentiation block was so severe that our criteria of dedifferentiation used above (i.e. connected cells at the hub with fragmented fusomes) was not applicable, although we frequently observed cysts with fragmenting fusomes, indicative of dedifferentiation. Twenty-nine percent of testes (*n*=45 testes) contained SGs but never progressed to SC differentiation (which can be recognized by growth in cell size) (Fig. 5B). In addition, 91% of testes (*n*=45 testes) contained SG cysts with ≥32 cells, further suggesting the failure in differentiation into SC stage (Fig. 5C,C′). It cannot be determined whether these SGs continue to proliferate (e.g. to 64 SG, 128 SG, etc.), as such cysts may also break apart by dedifferentiation. The fact that *nos* overexpression enhances *me31B*-knockdown phenotype implies that additional targets of *me31B* cooperate with misregulated *nos* to enhance the phenotype. Alternatively, further upregulation of endogenous *nos* due to *me31B* depletion and the *nos-tub3′UTR* transgene may enhance the effect.

### *nos* expression is dynamically regulated at multiple levels during differentiation in the male germline

Regulation of *nos* mRNA translation has been well documented and intensively studied, particularly in the context of germ cell specification (Gavis and Lehmann, 1992, 1994; Kugler and Lasko, 2009). The regulation of mRNA translation is critically important during oocyte development: the mRNAs that specify germ cell fate in the embryos, including *nos* and *osk* mRNA, are transcribed in nurse cells, transported into developing oocytes and stored in mature oocytes to be translated later (Lehmann, 2016). Accordingly, mRNA synthesis (transcription) is spatially and temporally separated from protein production (translation), making it critically important to control the timing of translation by both translational repression and activation.

**Fig. 6.**
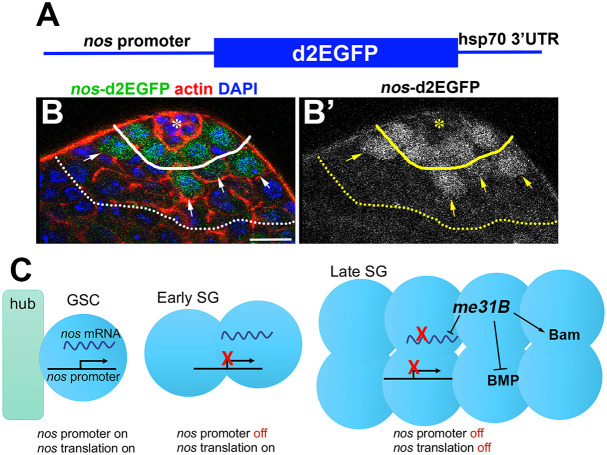
***nos* is transcriptionally and translationally regulated during *Drosophila* spermatogenesis.** (A) Diagram of the *nos* transcription reporter, in which the *nos* promoter drives unstable GFP protein and 3′ UTR sequence from *hsp70*, which makes mRNA short-lived. (B,B′) Apical tip of the testis expressing *nos* transcription reporter. The GSC-GB boundary is indicated by a solid line, and the four-cell/eight-cell SG boundary is indicated by the dotted line. GBs that are still connected to GSCs, thus still expressing *nos* transcription reporter, are indicated by arrows. Scale bar: 10 µm. The hub is indicated by the asterisk. (C) Model of *nos* regulation during germ cell development. In GSCs, the *nos* gene is transcribed and its mRNA is translated, leading to high Nos protein level and thus GSC maintenance. In early SGs, the *nos* gene is no longer transcribed, but Nos protein is produced via translation of inherited *nos* mRNA. In late SGs, the *nos* gene is no longer transcribed, and translation of *nos* mRNA is inhibited by *me31B*. This leads to a disappearance of Nos protein in these cells, promoting their differentiation. Interactions between *me31B* and its targets, indicated by arrows, may be direct or indirect.

Whether *nos* transcription is spatiotemporally distinct from Nos protein production during the development of male germ cells in the testis is not known. To address this question, we generated a *nos* promoter reporter by driving a destabilized GFP (d2EGFP) fused to the *hsp70 3′UTR* from the *nos* promoter (Fig. 6A). Because neither the mRNA nor protein products are stable in this reporter, the GFP signal closely recapitulates the activity of the *nos* promoter. Interestingly, we found that the *nos* promoter is active only in GSCs and GBs that are still connected to GSCs (Fig. 6B,B′), suggesting that *nos* is transcribed only in these early germ cells. These data suggest that Nos protein that is observed in two- to four-cell stage SGs is primarily produced by translation of *nos* mRNA inherited from GSCs and GBs (Fig. 6C). In addition, stable Nos protein generated in GSCs and GBs may contribute to its persistence through the four-cell SG stage.

These results reveal dynamic regulation of *nos* expression through multiple layers (Fig. 6C): (1) GSCs and GBs actively transcribe *nos* mRNA, which is translated to produce Nos protein; (2) two- and four-cell SGs no longer transcribe *nos* but inherit *nos* mRNA, which is translated to produce Nos protein; and (3) ≥eight-cell SGs do not transcribe *nos* mRNA, and translation of inherited *nos* mRNA is inhibited by Me31B, leading to overall downregulation of Nos protein. Loss of Me31B leads to increased translation of *nos* mRNA, thus increased levels of Nos protein, promoting dedifferentiation at later stages.

## DISCUSSION

Stem cell maintenance is critically important for long-term tissue homeostasis. Despite their ability to self-renew, stem cells are not immortal and their life span is often shorter than that of the organism. Dedifferentiation can replenish stem cell pools via conversion of more differentiated cells back into stem cell identity. However, uncontrolled dedifferentiation can lead to tumorigenesis (Landsberg et al., 2012; Schwitalla et al., 2013), thus proper control of dedifferentiation must be essential. Despite its importance, the mechanisms that regulate dedifferentiation are poorly understood.

This study identified *me31B* as a previously unknown and key negative regulator of dedifferentiation through its ability to regulate *nos* mRNA. Both *nos* and *bam* mRNAs co-immunoprecipitated with Me31B-GFP (Fig. 3G). Me31B may reinforce the known antagonistic relationship between *nos* and *bam* in the germline (Chen and McKearin, 2005; Li et al., 2009) by independently regulating these transcripts (Fig. 6C). In addition to extending Nos protein expression to eight-cell SGs and delaying Bam protein expression during germline development, depletion of *me31B* resulted in upregulation of BMP signaling, leading to an increased frequency of dedifferentiating SG cysts (Fig. 2). It remains unknown whether *me31B* directly regulates any components of BMP signaling. However, given the antagonistic relationship between *nos* and *bam*, and that BMP signaling represses *bam* expression (Chen and McKearin, 2003a,b, 2005; Harris et al., 2011; Li et al., 2012, 2009; Song et al., 2004; Wang and Lin, 2004), it is possible that BMP upregulation can be explained as a downstream effect of misregulated *nos* and/or *bam.*

In contrast to the deregulation of BMP signaling upon knockdown of *me31B*, STAT does not appear to be a relevant target of *me31B* in inducing dedifferentiation (Fig. S2). *bam>me31B^RNAi^* testes did not detectably alter STAT signaling. Importantly, when a cyst of dedifferentiating *bam>me31B^RNAi^* SGs was attached to the hub cells, only the germ cells that were in direct contact with the hub had high STAT levels (Fig. S2B, arrow). These results indicate that germ cells in ≥four-cell SG cysts can reestablish STAT signaling upon homing into the niche during dedifferentiation triggered by depletion of *me31B*. Although downregulation of JAK-STAT signaling is reported to prevent SG dedifferentiation (Sheng et al., 2009), our data suggest that the dedifferentiation induced by depletion of *me31B* does not directly involve the activation of the JAK-STAT pathway. We speculate that JAK-STAT signaling might help maintain GSCs that were generated by dedifferentiation, instead of inducing dedifferentiation per se. Interestingly, however, STAT expression was reduced in GSCs of the *nos>me31B^RNAi^* testes compared to controls (Fig. S2C-D″), suggesting that *me31B* has an additional role in GSCs to maintain STAT activation. Reduced STAT in *nos>me31B^RNAi^* testes, which may deplete native GSCs, might explain the higher frequency of dedifferentiation with *nos-gal4-*driven *me31B^RNAi^* compared to *bam-gal4*-driven *me31B^RNAi^* (Fig. 1F).

It remains elusive what controls *me31B* to promote differentiation and/or prevent dedifferentiation. Is *me31B* downregulated by conditions that trigger dedifferentiation? We did not observe any changes in Me31B-GFP protein level or localization when dedifferentiation was artificially induced by transient expression of Bam (not shown). In future studies, it will be of interest to investigate whether and how Me31B senses niche vacancy (missing GSCs) to trigger dedifferentiation of SGs.

The right balance of differentiation and dedifferentiation must be achieved to ensure maintenance of the stem cell pool, while minimizing the risk of tumorigenesis. The results presented in this study suggest that SGs are in a state of transition from stem cell identity to full commitment to differentiation (SC). Whereas GSCs produce Nos protein via *nos* mRNA transcription and its translation, two- and four-cell SGs produce Nos protein only via translation of inherited *nos* mRNA. We propose that two- and four-cell SGs represent a critical cell population/developmental time window that is not yet fully committed to differentiation but maintains the potential to dedifferentiate, as they still have Nos protein like GSCs, but no longer transcribe *nos* unlike GSCs (Fig. 6C). These SGs may hit a perfect balance of Nos protein that maintains their potential to dedifferentiate into GSCs as necessary, but prevents tumorigenesis by shutting down *nos* transcription. Indeed, two- and four-cell SGs are known to be most potent for dedifferentiation (Sheng and Matunis, 2011): although this was speculated to be mostly due to their physical proximity to the hub cells, it is also possible that their ‘Nos production state’ (actively producing Nos protein from inherited mRNA) is more suited for dedifferentiation than later SGs. We propose that stepwise transitions from the stem cell state to the differentiated state are key for maintaining the stem cell pool while preventing tumorigenesis. In summary, the present study provides a new insight into how gradual commitment to differentiation is ensured by transcriptional and translational control of a key regulator of cell fate.

## MATERIALS AND METHODS

### Fly husbandry and strains

Unless otherwise stated, all flies were raised on standard Bloomington medium at 25°C, and young flies (1- to 3-day-old adults) were used for all experiments. See Table S1 for the list of stocks used in this study.

### Immunofluorescence staining and microscopy

Immunofluorescence staining was performed as described previously (Cheng et al., 2008). Briefly, tissues were dissected in PBS, transferred to 4% formaldehyde in PBS and fixed for 30 min. Tissues were then washed in PBS-T (PBS containing 0.1% Triton X-100) for at least 30 min (three 10 min washes), followed by incubation with primary antibody in 3% bovine serum albumin (BSA) in PBS-T at 4°C overnight. Samples were washed for 60 min (three 20 min washes) in PBS-T, incubated with secondary antibody in 3% BSA in PBS-T at 4°C overnight, washed as above, and mounted in Vectashield with DAPI (Vector Labs). The antibodies used are described in Table S2. Images were taken using a Leica TCS SP8 confocal microscope with 63× oil-immersion objectives (NA=1.4). Images were processed using Adobe Photoshop and ImageJ software.

Dedifferentiating SG cysts were identified as the cysts containing ≥3 SGs that are connected to each other by the fragmented fusome (Fig. 1C,D). In contrast, normally differentiating SGs contain continuous fusome that connects all cells within the cyst (Fig. 1E). Thus, the morphology of the fusome distinguishes differentiating SGs versus dedifferentiating SGs. The connectivity of cells within the dedifferentiating SGs was determined by the presence of fusome fragments between two cells within a cyst: for example, the connection between cell 1 and cell 2 can be confirmed by the presence of the fusome fragment between these two cells. Cell 2 may be then connected to cell 3 with another fragment of the fusome, establishing the connectivity of cell 1, 2 and 3, and so on. In rare cases, when two cells clearly shared the cytoplasm by continuous Vasa staining, such cell pairs may be determined as connected without the presence of fusome in between. When ≥3 cells were determined to be connected to each other with this method, and found at the hub cells, such SG cysts were scored as ‘dedifferentiating’. Significance was determined using a Fischer's Exact Test in comparison to a control.

### RNA Fluorescent *in situ* hybridization

To detect *nos* mRNA, single molecule fluorescent *in situ* hybridization (smFISH) was conducted by following a previously described protocol (Fingerhut et al., 2019). All solutions used for smFISH were RNase free. Testes from 2-3-day-old flies were dissected in 1× PBS and fixed in 4% formaldehyde in 1× PBS for 30 min. Then testes were washed briefly in PBS before being rinsed with wash buffer [2× saline-sodium citrate (SSC) and 10% formamide] and then hybridized overnight at 37°C in hybridization buffer [2× SSC, 10% dextran sulfate (Sigma-Aldrich, D8906), 1 mg ml^−1^ *E. coli* tRNA (Sigma-Aldrich, R8759), 2 mM vanadyl ribonucleoside complex (New England Biolabs, S142) and 0.5% BSA (Ambion, AM2618), 10% formamide]. Following hybridization, samples were washed three times in wash buffer for 20 min each at 37°C and mounted in Vectashield with DAPI (Vector Labs). Images were acquired using an upright Leica TCS SP8 confocal microscope with a 63× oil immersion objective lens (NA=1.4) and processed using Adobe Photoshop and ImageJ software. Fluorescently labeled probes were added to the hybridization buffer to a final concentration of 50 nM (for satellite DNA transcript targeted probes). Probe set against *nos* exons was designed using the Stellaris RNA FISH Probe Designer (Biosearch Technologies) available online at www.biosearchtech.com/stellarisdesigner. The Stellaris RNA FISH (Biosearch Technologies) probes were labeled with Quasar 670. Probe set was added to the hybridization buffer at a 50 nM final concentration. For smFISH probe sequences, see Table S3.

### RNA immunoprecipitation-qPCR

Samples were collected from two genotypes, a control (*nos-gal4>UAS-GFP, UAS-dpp*) and an experimental (*nos-gal4>UAS-dpp, me31B-GFP*), and processed in pairs. Dpp overexpression (*UAS-dpp*) was introduced to increase SGs in the sample. Approximately 200 testes per sample were collected into RNase-free PBS, frozen in liquid nitrogen after removing excess liquid, and stored at −80°C until extraction. Lysis was completed by grinding the tissue in 400 µl of lysis buffer [150 mM KCl, 20 mM HEPES (pH 7.4), 1 mM MgCl_2_ with 1× cOmplete EDTA-free Protease Inhibitor Cocktail and 1 U μl^−1^ RNasin Plus RNase Inhibitor from Promega added right before the use] and incubating for 30 min on ice with pipetting every 10 min. After centrifugation at 12,000 ***g*** for 5 min, pelleted cell debris were discarded. At this point, a 10% pre-immunoprecipitation input sample was removed and saved to serve as a control. For precipitation of Me31B-GFP and control GFP, GFP-conjugated magnetic beads were prepared by incubating 10 µg of mouse anti-GFP antibodies (Fisher Scientific) with 50 µl of Protein G Dynabeads in 200 µl of Ab Binding and Washing Buffer (provided in the kit) for 10 min at room temperature on a rotator. After antibody conjugation, beads were magnetically separated and washed once with 200 µl of Ab Binding and Washing Buffer. The antibody-conjugated beads were then incubated with the lysate for 10 min at room temperature (samples tubes were tumbled end-over-end during incubation). After magnetic separation of the beads, 10% of the supernatant was taken as non-bound fraction sample. The beads were washed with the Dynabeads Protein G Kit Washing Buffer three times, and were resuspended in TRIzol (the 10% pre-immunoprecipitation and 10% post-immunoprecipitation samples were also processed with TRIzol at this time) according to the manufacturer's instructions. cDNA was generated using SuperScript III Reverse Transcriptase (Invitrogen) followed by qPCR using Power SYBR Green reagent (Applied Biosystems). Inputs of 10% were diluted to a 1% input before reverse transcription was run. The fold enrichment was calculated by the ΔΔCt method. First, Ct values from each immunoprecipitation sample were normalized to their respective 1% input for each primer (ΔCt) to account for RNA sample preparation differences:
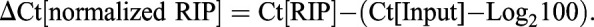


Then, the ΔΔCt (Me31B-GFP/control GFP) was obtained to compare these normalized values between the Me31B-GFP sample versus the UAS-GFP control for each primer set:
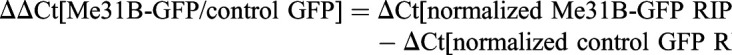


Finally, the fold enrichment was obtained using the following formula:
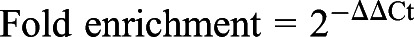
Experiments were performed in technical triplicates with three biological replicates. The following primers were used: *rp49*, forward 5′-TACAGGCCCAAGATCGTGAA-3′, reverse 5′-TCTCCTTGCGCTTCTTGGA-3′; *nos* set #1, forward 5′-CAGTACCACTACCACTTGCTG-3′, reverse 5′-AAAGATTTTCAAGGATCGCGC-3′; *nos* set #2, forward 5′-CACCGCCAATTCGCTCCTTAT-3′, reverse 5′-GCTGGTGACTCGCACTAGC-3′; and *bam*, forward 5′-TGACGTTACTGCACCACTCC-3′, reverse 5′-CGAACAGATAGTCCGAGGGC-3′.

## Supplementary Material

Supplementary information

Reviewer comments
