# Supplementary material for: me31B regulates stem cell homeostasis by preventing excess dedifferentiation in the Drosophila male germline
Source: J Cell Sci. 2021 Jul 22;134(14):jcs258757. doi: 10.1242/jcs.258757 (PMC8325955; doi:10.1242/jcs.258757)
Supplement: Supplementary information [file joces-134-258757-s1.pdf]

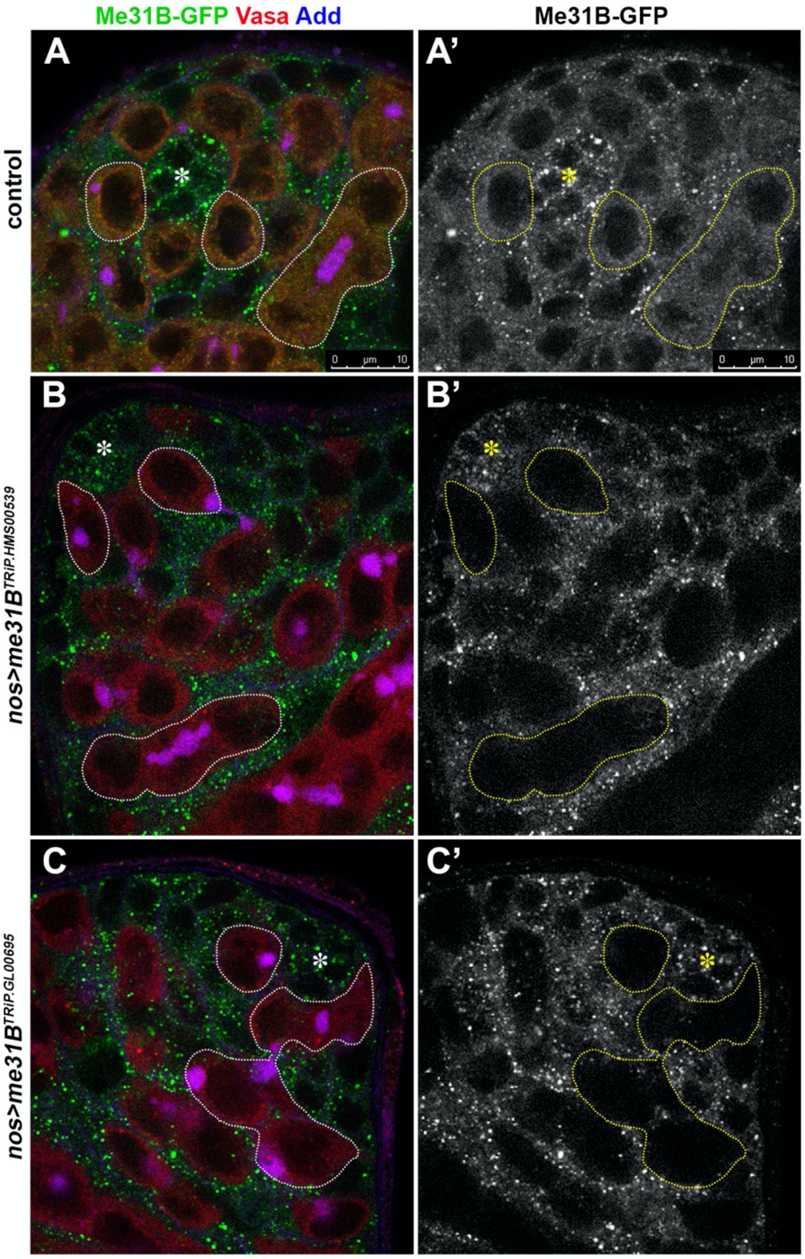

Figure S1. Germline-specific knockdown of *me31B*. Two independent *me31B* knockdown constructs were expressed using the *nos-gal4* driver in the Me31B-GFP protein trap line. GFP signal was diminished in germ cells upon expression of *me31B* knockdown constructs, leaving the GFP signal in the somatic cyst cells.

A. control testis, B. *nos>me31B*^{TRiPHMS00539}, C. *nos>me31B*$^{TRiP.GL00695}$. Examples of germ cell cysts are indicated by dotted lines. The hub is indicated by asterisks. Red: Vasa (germ cells, nuage), Blue: Adducin-like (Add, fusome, note that Blue appears to be magenta in the figure due to overlap with Vasa signal in red), Green: Me31B-GFP

Journal of Cell Science • Supplementary information

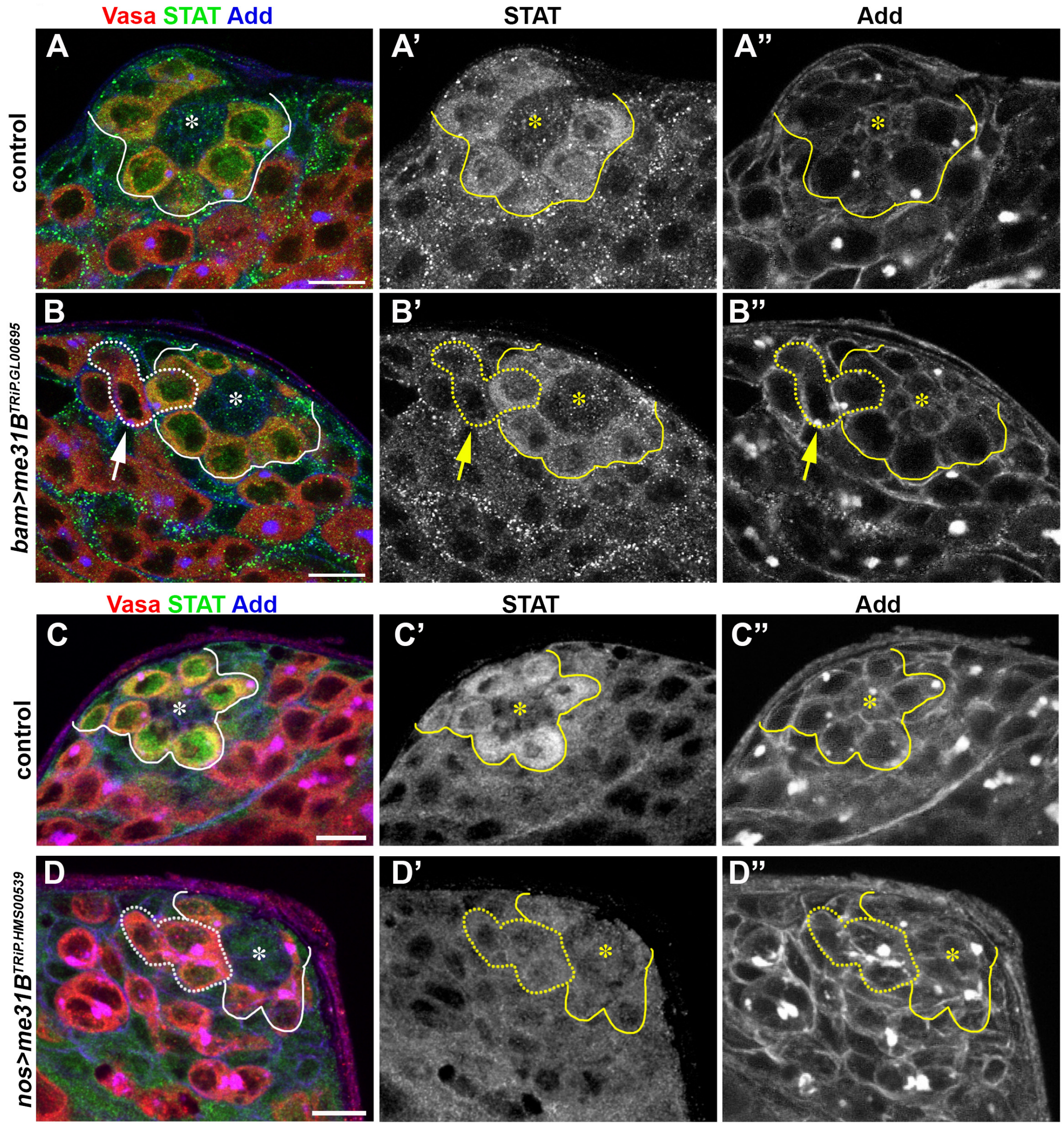

Figure S2. STAT expression upon *me31B*$^{TRiP.GL00695}$.

A-B. STAT signal in GSCs was reduced upon RNAi-mediated knockdown of *me31B* by *bam-gal4* driver. Control (A), and *bam>me31B*$^{TRiP.GL00695}$ (B) testes. In B, a dedifferentiating cyst is indicated by the arrow, where only the germ cell that is attached to the hub has a high STAT signal, whereas the remaining germ cells do not have a high STAT signal. STAT level was monitored by anti-STAT antibody. GSCs are indicated by solid line. Dedifferentiating cysts, identified by fragmented fusomes connecting ≥3 germ cells and attachment to the hub, are indicated by dotted line. Hub is indicated by asterisk. Bar: 10μm. n= 47 for control, n= 44 for *bam>me31B*$^{TRiP.GL00695}$, n= 39 for *bam>me31B*$^{TRiP.HMS00539}$ (100% of testes exhibited normal STAT in all genotypes)
C-D. STAT signal did not change upon RNAi-mediated knockdown of *me31B* by *nos-gal4* driver. Control (C) and *nos>me31B*$^{TRiP.HMS00539}$ (D) testes. n= 10 for control, n= 36 for *nos>me31B*$^{TRiP.GL00695}$(81% of testes exhibited downregulated STAT), n= 17 for *nos>me31B*$^{TRiP.HMS00539}$ (100% of testes exhibited downregulated STAT).

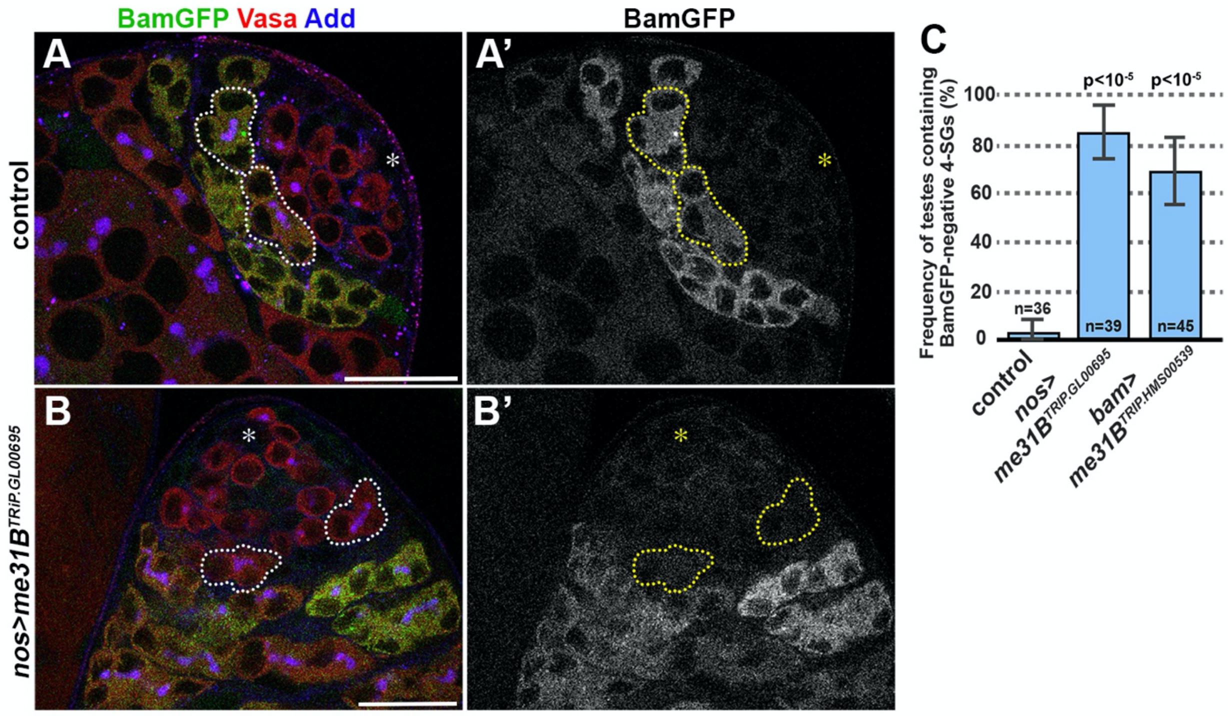

Figure S3. Bam expression is delayed upon knockdown of *me31B*.
A. In control testis, germ cells start expressing Bam-GFP in 4-cell SG stage (indicated by dotted lines). B. Upon knockdown of *me31B*, 4-cell SGs often lacks Bam-GFP expression (dotted lines). Hub is indicated by asterisk. Bar: 25µm. C. Frequency of testes containing 4-cell SG without Bam expression in control vs. *me31B*^{RNAi} testes. n = number of testes scored. p-value from the Fisher's exact test is provided.

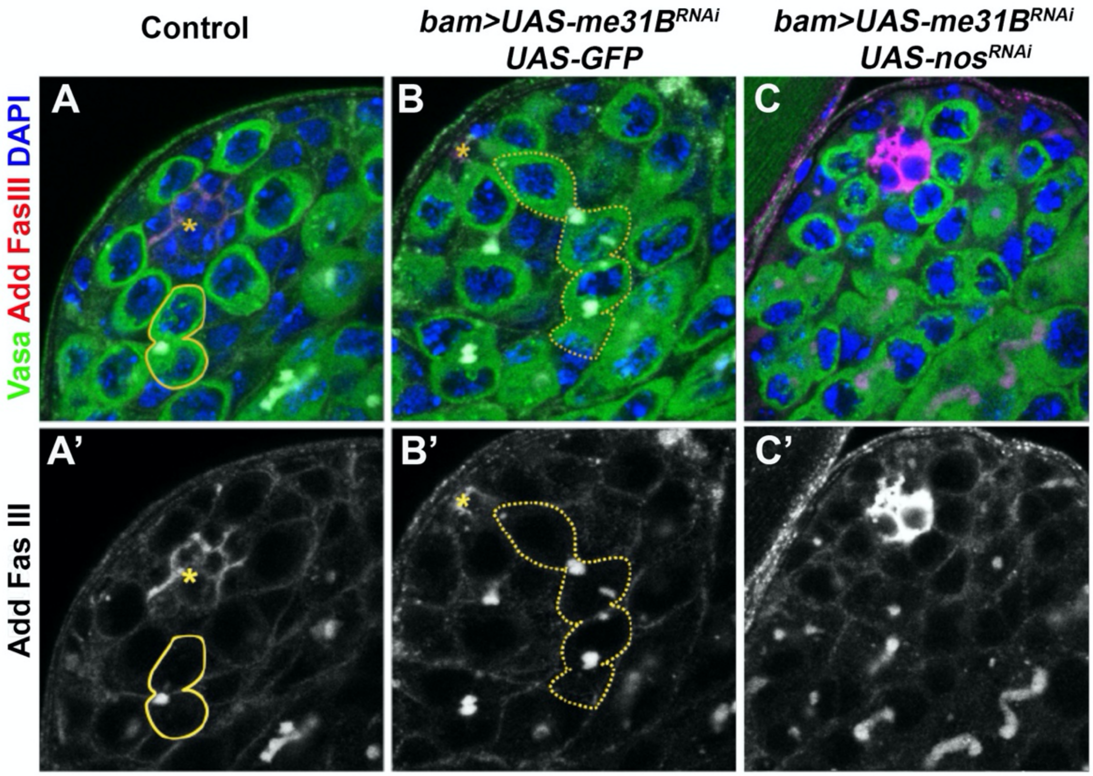

Figure S4. *nos* is required for spermatogonial dedifferentiation induced by *me31B* depletion.

A. Control, B. *bam>UAS-me31B*^{RNAi}*, UAS-GFP. UAS-GFP* was expressed to control for the number of transgenes driven by bam-gal4. C. *bam>UAS-me31B*^{RNAi}*, UAS-nos*^{RNAi}. *nos* depletion prevented dedifferentiation induced by *me31B*^{RNAi}.

Journal of Cell Science • Supplementary information

Table S1. list of Drosophila stocks in this study

Symbol/name used in publication	Source information
nos-gal4 (on Chr2)	PMID: 9501989
nos-gal4 (on Chr3)	FBti0012410/ PMID: 9501989
bam-gal4	PMID: 12571107, Gift from Dennis McKearin
UAS-me31B P{TRiP.HMS00539}attP2	FBst0033675
UAS-me31B P{TRiP.GL00695}attP40	FBst0038923
UAS-me31B P{TRiP.HM05052}attP2	FBst0028566
*UAS-tkv**	FBst0036537
STAT-GFP	FBst0038670
nos-GFP	FBal0339106
hs-FLP, nos-FRT-stop-FRT-gal4	PMID: 24465278
nos-dE2EGFP	see methods
UAS-nos-tub3'UTR	FBal0141015/ PMID: 12091303
UAS-nos P{TRiP.JF02931}attP2	FBst0028300
UAS-nosP{TRiP.HMS00785}attP2	FBst0032985
UAS-nosP{TRiP.HMS00930}attP2	FBst0033973
Me31B-GFP	FBst0051530
Bam-GFP	FBal0144433/ PMID: 12571107
UAS-GFP	FBti0013987
UAS-Dpp	FBst0001486

Table S2. list of antibodies used in this study

Anti-pSmad	Cell Signaling: Phospho-Smad1/5 (Ser463/465) (41D10) Rabbit mAb #9516	1:100 dilution for immunofluorescence (IF) staining
Anti-GFP	Fisher Scientific, mouse monoclonal antibody (3E6)	Used for RIP (see methods)
Anti-STAT	PMID: 26131929	1: 5000 dilution (from original serum)
Anti-Add/Hts	DSHB	1:20 dilution for IF
Anti-Vasa	DSHB	1:20 dilution for IF

Table S3. List of probe sequences for nos RNA in situ probes (Stellaris ®). Probes were conjugated to Quasar 670.

5’-tccaagttgctgcggaacat-3’/ 5’-aaagttatctgctgctgcgc-3’/ 5’-ctcctctggcgtgaaaagca-3’/ 5’-tgcaggcccagaatgttgag-3’/ 5’-ccactggtatccaaatacat-3’/ 5’-gtaatgggcggactcaaagt-3’/ 5’-tcggccagaaaagggaagtg-3’/ 5’-cataaggagcgaattggcgg-3’/ 5’-caagtggtagtggtactgtc-3’/ 5’-ttgctggtgactcgcactag-3’/ 5’-aaggatcgcgcaatctcgtc-3’/ 5’-cgtcacctgcgcaaagattt-3’/ 5’-catagccattggtcgcgaac-3’/ 5’-taggacatgcgaccgagatc-3’/ 5’-cattaagttgccgccattgg-3’/ 5’-agtgggtggcgagtggaatg-3’/ 5’-cacacgttgttcagatgctc-3’/ 5’-ggctggtatatacgacatgt-3’/ 5’-ctgcaaacccattgtattgg-3’/ 5’-cgagattggtggacacagtg-3’/ 5’-tactggaattggaagctccg-3’/ 5’-ttgctgttgtaacgcttgta-3’/ 5’-aaaagacgcagtggcggctg-3’/ 5’-tctggttcgttgttattctc-3’/ 5’-gcactgagtggctattgata-3’/ 5’-cacagcactcggttaaagtt-3’/ 5’-acacgtaggtgcgtagtttg-3’/ 5’-cagtacttaatcgtgtgcgc-3’/ 5’-atggtgatgatcggcttctt-3’/ 5’-gaacgattccgccttgatcg-3’/ 5’-agtaactgctcttggctagg-3’/ 5’-taaaccttcatctgttgctt-3’

Journal of Cell Science • Supplementary information
