# Supplementary material for: me31B regulates stem cell homeostasis by preventing excess dedifferentiation in the Drosophila male germline
Source: J Cell Sci. 2021 Jul 22;134(14):jcs258757. doi: 10.1242/jcs.258757 (PMC8325955; doi:10.1242/jcs.258757)
Supplement: Reviewer comments [file joces-134-258757_review_history.pdf]

Journal of Cell Science

me31B regulates stem cell homeostasis by preventing excess dedifferentiation in the *Drosophila* male germline

Lindy Jensen, Zsolt G Venkei, George Watase, Bitarka Bisai, Scott Pletcher, Cheng-Yu Lee and Yukiko M Yamashita

DOI: 10.1242/jcs.258757

Editor: David Glover

Review timeline

Original submission:	7 April 2021
Editorial decision:	10 May 2021
First revision received:	1 June 2021
Accepted:	14 June 2021

Original submission

First decision letter

MS ID#: JOCES/2021/258757

MS TITLE: me31B regulates stem cell homeostasis by preventing excess dedifferentiation in the Drosophila male germline.

AUTHORS: Lindy Jensen, Zsolt G Venkei, George Watase, Bitarka Bisai, Scott Pletcher, Cheng-Yu Lee, and Yukiko M Yamashita
ARTICLE TYPE: Research Article

We have now reached a decision on the above manuscript.

To see the reviewers' reports and a copy of this decision letter, please go to: https://submit-jcs.biologists.org and click on the 'Manuscripts with Decisions' queue in the Author Area. (Corresponding author only has access to reviews.)

As you will see, the reviewers raise a number of substantial criticisms that prevent me from accepting the paper at this stage. They suggest, however, that a revised version might prove acceptable, if you can address their concerns. If you think that you can deal satisfactorily with the criticisms on revision, I would be pleased to see a revised manuscript. We would then return it to the reviewers.

We are aware that you may be experiencing disruption to the normal running of your lab that makes experimental revisions challenging. If it would be helpful, we encourage you to contact us to discuss your revision in greater detail. Please send us a point-by-point response indicating where you are able to address concerns raised (either experimentally or by changes to the text) and where you will not be able to do so within the normal timeframe of a revision. We will then provide further guidance. Please also note that we are happy to extend revision timeframes as necessary.

Please ensure that you clearly highlight all changes made in the revised manuscript. Please avoid using 'Tracked changes' in Word files as these are lost in PDF conversion.

© 2021. Published by The Company of Biologists under the terms of the Creative Commons Attribution License (https://creativecommons.org/licenses/by/4.0/).

I should be grateful if you would also provide a point-by-point response detailing how you have dealt with the points raised by the reviewers in the 'Response to Reviewers' box. Please attend to all of the reviewers' comments. If you do not agree with any of their criticisms or suggestions please explain clearly why this is so.

Reviewer 1

Advance summary and potential significance to field

Stem cell progeny close to self renewing signal still manage to differentiated efficiently. How these stem cell progeny differentiate instead of dedifferentiating is not fully understood. Jensen et al. describe the role of me31B in regulating male germline stem cell (GSC) differentiation by negatively regulating dedifferentation. In response to a loss of GSCs, differentiating cells can dedifferentiate, however, it was previously not known how dedifferentiation is regulated. Me31B is a translational regulator that is known to play a key role in germline development, but it had not been implicated as a regulator of dedifferentiation. By using fragmented fusomes as a marker of dedifferentiation, the authors implicate that Me31B is an important regulator that prevents excess dedifferentiation under wild type conditions. The authors find that these dedifferentiating cells express the stem cell marker pMad indicating that these cysts revert fate. Mechanistically, the authors demonstrate that me31B is required for the proper expression of both Nos, a protein implicated in stem cell maintenance, and Bam, a master regulator of differentiation. Additionally, the authors find that me31B physically interacts with these mRNAs. Furthermore, they find that the depletion of nos can rescue the me31B dedifferentiation phenotype, while expressing an unregulated nos transgene (which has a replaced 3'UTR), phenocopies loss of me31b. This is consistent with a model where me31b negatively regulates nos translation and limits Nos expression preventing dedifferentiation. Thus, these finding implicate translational control as a barrier to dedifferentiation.

Comments for the author

The authors present a careful and interesting study that implicates me31B as a regulator of Nos expression and Nos as a regulator of GSC dedifferentiation, there are certain concerns that need to be addressed to ascertain these findings:

1. The authors report that loss of me31B results in dedifferentiation using fragmented fusome morphology as a marker of this process. The authors cite previous research to support their use of this marker. However previous research has mostly focused on dedifferentiation under normal conditions or under conditions where differentiation has been upregulated by overexpression of Bam. As the authors are depleting a known regulator of differentiation, me31B, it is necessary to show that these cells are do not have a cytokinesis defect during differentiation.

2. Throughout the paper the authors outline cells to highlight cells with fusome breakdown. However, in some instances it is unclear whether fusome breakdown is taking place as it is unclear whether the highlighted cells are actually connected such as in Figure 1D, 2C. They can supplement a few critical observation with p-Try staining to show that these cells are in fact connected.

3. The authors state that they observe a reduction of STAT in dedifferentiating cysts depleted of me31b using nosGal4 as a driver, but not when using bamGal4 as a driver. However, with nosGal4 the authors use a STAT-GFP line, while with bamGal4 the authors use a STAT antibody, precluding comparison between the two conditions. Can the authors use the same reagent in both? If they cannot, they should state the caveat in the text. Additionally, it is not readily apparent to this reviewer that the cells surrounded by a dotted outline in Supplemental Figure S2D are in fact connected (the last cell in the yellow dotted line area).

4. If comparing RNA in situ levels and saying that loss of Me31B does not affect mRNA levels of nos, there should be quantification showing that there is no difference in RNA levels. In F and F' there appears to be higher levels of RNA in one of the wild type cysts when compared to the clone. Additionally, the size of the scale bars in Figure 3E-F are not stated.

5. Figure 3G lacks error bars. Additionally, according to the methods this IP experiment was performed from a NosGAL4>UAS-dpp background to enrich for SCs. This should be stated in the text

© 2021. Published by The Company of Biologists under the terms of the Creative Commons Attribution License (https://creativecommons.org/licenses/by/4.0/).

to enhance reader clarity and prior literature cited to support this approach. Further, the observation that me31B binds to bam RNA should be contextualized as likely occurring prior to Bam protein expression and differentiation. Finally the authors should address the confounding nature of these results. Me31b is canonically a translational repressor, yet the authors show that loss of Me31B results in a delay in bam expression, despite the direct association between bam RNA and Me31b.

6. The authors report that me31B knockdown leads to delayed Bam expression (Figure 3S). However, the authors use a version of Bam-GFP that has a functional bam promoter controlling expression of GFP-tagged Bam, but lacks the 5'UTR (at least according to the stock number listed). This version of Bam-GFP has been reported to express in GSCs in ovaries, however, this is not apparent from this study. It is unclear to this reviewer what this experiment is meant to demonstrate using this line.

7. The authors report that simultaneous upregulation of nos and me31B knockdown results in >32 cell SGs.
This seems consistent with a differentiation defect rather than what would be expected by the authors proposed model which would be an excess of undifferentiated cells or dedifferentiating cells.

8. The authors report that simultaneous knockdown of me31B and nos greatly reduces the amount of germaria that contain dedifferentiating cysts. It would be helpful to the reader to see a representative image.

9. The scale bar is different in Figure 1E and E', size of scale bar should be stated in the legends.

10. The model in figure 6 suggests that me31B directly activates bam expression. The model could be misleading to a reader by showing an arrow as it may indicate that Me31B directly activates Bam translation.
Additionally, the model represents both nos transcription and translation, however, the data presented do not differentiate between the two.

11. In line 321-323 it states, "Overall, we conclude that me31B prevents dedifferentiation of SGs by reducing Nos protein levels and increasing Bam protein levels." However, the authors have not demonstrated that me31B directly increases bam protein levels.

<u>Reviewer 2</u>

Advance summary and potential significance to field

The paper by Jensen et al builds a compelling case that Me31B-mediated translational repression of Nos regulates de-differentiation of spermatogonia in the Drosophila testis, and is thus critical to establishing and maintaining stem cell homeostasis. They also demonstrate that Me31B knockdown leads to hyperactivation of BMP signaling in germ cells, and that de-repression of Nos is sufficient for spermatogonial de-differentiation. Exactly how Me31B and Nos may regulate BMP signaling remains unclear. Nevertheless, the paper concludes with an interesting model, that 4- and 8-cell spermatogonia are delicately balanced as to whether to further differentiate or to de-differentiate, because they no longer transcribe nos RNA but are still able to translate it. In summary, I believe this manuscript makes a substantial and highly significant contribution to our understanding of molecular mechanisms underlying stem cell homeostasis.

Comments for the author

I believe the paper only requires minor revisions prior to publication. Most substantively, I would like to have more information about the striking differentiation block that occurs upon knockdown of me31B and overexpression of nos. In particular, what happens to the cysts that have undergone one or more additional mitotic divisions? Do they try to differentiate into spermatocytes and express markers for later stages of development, or do they just undergo cell death shortly after the developmental stage shown in Figure 5C?

© 2021. Published by The Company of Biologists under the terms of the Creative Commons Attribution License (https://creativecommons.org/licenses/by/4.0/).

While not required in my view for this paper, further analysis of gene expression in these testes may identify additional Nos and Me31B targets with important roles in later cell differentiation events.

I also have some small presentational comments.

Lines 36 and 39: Two consecutive sentences should not start with 'However...'; this is confusing.

Line 248: 'of' is missing between 'deregulation' and 'STAT'.

Line 255: 'of' is missing between 'activation' and 'STAT'.

Lines 253-257: I think this material is more suitable for the discussion than for the results. The authors should also consider a different balance between the presentation of the somewhat inconclusive results with STAT, that are in a supplemental figure, and the clear results with BMP, that are in a main figure. I think it would be better to present the BMP results first and then the STAT results more briefly.

Line 292: should read 'testes' not 'testis'

Line 321: I think bam RNA should be described as a 'potential additional target' for Me31B as there is only the RIP-qPCR evidence that supports this. In vivo effects could be indirect through Nos.

Line 390: replace 'to' with 'do'.

Line 409: Insert 'in the' between 'bam' and 'germline'.

First revision

<u>Author response to reviewers' comments</u>

Reviewer 1 Advance Summary and Potential Significance to Field:
Stem cell progeny close to self renewing signal still manage to differentiated efficiently. How these stem cell progeny differentiate instead of dedifferentiating is not fully understood. Jensen et al. describe the role of me31B in regulating male germline stem cell (GSC) differentiation by negatively regulating dedifferentation. In response to a loss of GSCs, differentiating cells can dedifferentiate, however, it was previously not known how dedifferentiation is regulated. Me31B is a translational regulator that is known to play a key role in germline development, but it had not been implicated as a regulator of dedifferentiation. By using fragmented fusomes as a marker of dedifferentiation, the authors implicate that Me31B is an important regulator that prevents excess dedifferentiation under wild type conditions. The authors find that these dedifferentiating cells express the stem cell marker pMad indicating that these cysts revert fate. Mechanistically, the authors demonstrate that me31B is required for the proper expression of both Nos, a protein implicated in stem cell maintenance, and Bam, a master regulator of differentiation. Additionally, the authors find that me31B physically interacts with these mRNAs. Furthermore, they find that the depletion of nos can rescue the me31B dedifferentiation phenotype, while expressing an unregulated nos transgene (which has a replaced 3'UTR), phenocopies loss of me31b. This is consistent with a model where me31b negatively regulates nos translation and limits Nos expression preventing dedifferentiation. Thus, these finding implicate translational control as a barrier to dedifferentiation.

Reviewer 1 Comments for the Author:
The authors present a careful and interesting study that implicates me31B as a regulator of Nos expression and Nos as a regulator of GSC dedifferentiation, there are certain concerns that need to be addressed to ascertain these findings:

© 2021. Published by The Company of Biologists under the terms of the Creative Commons Attribution License (https://creativecommons.org/licenses/by/4.0/).

Thank you for going through the manuscript so thoroughly, and catching mistakes in the manuscript. We have addressed this reviewer's concern as detailed below. Hope that these textual changes and added data/new images satisfactorily address this reviewer's concerns.

1. The authors report that loss of me31B results in dedifferentiation using fragmented fusome morphology as a marker of this process. The authors cite previous research to support their use of this marker. However, previous research has mostly focused on dedifferentiation under normal conditions or under conditions where differentiation has been upregulated by overexpression of Bam. As the authors are depleting a known regulator of differentiation, me31B, it is necessary to show that these cells are do not have a cytokinesis defect during differentiation.

We appreciate this reviewer for bringing this up. This is one of frequently asked questions on this work---how do we know that me31B-depleted germ cells are dedifferentiating, instead of failing cytokinesis or differentiating right at the hub. Let us first clarify the definition of 'cytokinesis defect'. Clearly, this reviewer is not referring to the total failure of cytokinesis, which would result in multi-nucleated cells. Instead, judging from the context, this reviewer is referring to 'incomplete cytokinesis', which is observed in SGs during normal differentiation. Thus, this reviewer's question is 'how do we know that me31B-depleted cells are not just undergoing differentiation and halting cytokinesis as normal SGs would do? What if our observation is not pointing toward dedifferentiation at all?'

There are two reasons that strongly argue against these possibilities.
First, we observe the cyst containing ≥3 SGs ('3+ SGs' for short) at the hub cells (stem cell position) even when me31B is depleted by bam-gal4 driver. Under this condition, GSCs are **NOT** depleted of me31B, and there is no reason for normal GSCs to 'fail to complete cytokinesis'. 3+ SGs at the hub observed in bam>me31B-RNAi must be due to depletion of me31B in bam+ cells (i.e. 4-SG or later). This supports the notion that 3+SGs observed upon me31B-depletion is caused by dedifferentiation of SGs, instead of incomplete cytokinesis of GSCs/GBs.

Second, differentiating SGs and dedifferentiating SGs can be distinguished by the morphology of fusome as detailed in the text. As Figure 1E shows, normally-differentiating SGs contain continuous fusome. On the contrary, dedifferentiating SGs contain fragmented fusome, which only connects two cells within the SG cyst. Such morphology is specifically observed during dedifferentiation.

Taken together, we believe that incomplete cytokinesis is an unlikely explanation for our observation. We have revised the text (with added explanation of identifying dedifferentiating SGs in the method section) to clarify these points.

2. Throughout the paper the authors outline cells to highlight cells with fusome breakdown. However, in some instances it is unclear whether fusome breakdown is taking place as it is unclear whether the highlighted cells are actually connected such as in Figure 1D, 2C. They can supplement a few critical observation with p-Try staining to show that these cells are in fact connected.

We understand this reviewer's concern, but p-Tyr will not work for this purpose. It has been shown by Matunis' group (Brawly et al.) that ring canal also disintegrates during dedifferentiation and therefore, p-Tyr or any other ring canal markers will not mark all connected cells. However, we believe that our identification of connected cells is quite unambiguous. This is explained in the text (with edits and addition to method section): to elaborate, connectivity of two neighboring cells were always confirmed by the fusome. As shown in the below image (these are from Figure 1C and D), the connectivity of two neighboring cells was always ensured by the fusome that bridges two cells. In this manner, we can track all the cells within the same cyst. Even though the fusome breaks down during dedifferentiation, we used remaining fusome that are still clearly bridging two cells to make a call of which cells are connected. Alternatively, in very rare cases when two cells are so clearly connected through their cytoplasm, we scored them as connected even if fusome was not clearly bridging them (example is seen in Figure S2D).

Together, we're fairly confident that our identification of dedifferentiating cysts is accurate---except that we're most likely under-estimating the occurrence of dedifferentiation because we do not score SGs as dedifferentiating if the fusome is already gone.

© 2021. Published by The Company of Biologists under the terms of the Creative Commons Attribution License (https://creativecommons.org/licenses/by/4.0/).

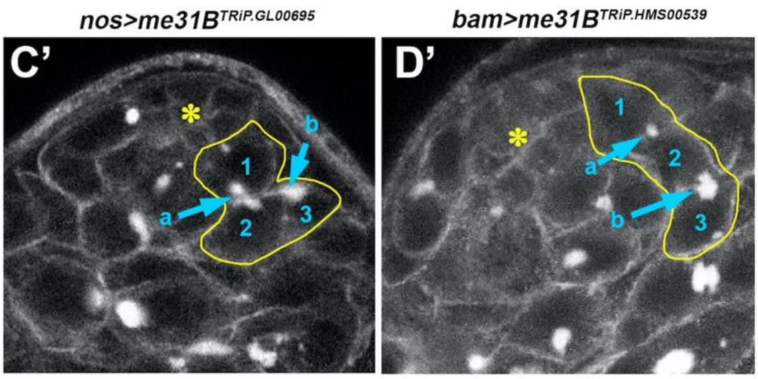

In these panels, cell 1 and cell 2 is connected by fusome a, and cell 2 and cell 3 are connected by fusome b.

3. The authors state that they observe a reduction of STAT in dedifferentiating cysts depleted of me31b using nosGal4 as a driver, but not when using bamGal4 as a driver. However, with nosGal4 the authors use a STAT-GFP line, while with bamGal4 the authors use a STAT antibody, precluding comparison between the two conditions. Can the authors use the same reagent in both? If they cannot, they should state the caveat in the text. Additionally, it is not readily apparent to this reviewer that the cells surrounded by a dotted outline in Supplemental Figure S2D are in fact connected (the last cell in the yellow dotted line area).

STAT-GFP and anti-STAT staining have been used interchangeably in the field, and indeed original Figure S2A and S2C (STAT-GFP and anti-STAT, both in control genotype) show that these two methods are comparable. Our use of STAT-GFP vs. anti-STAT was mostly because of the convenience of genetics, but we replaced all images with anti-STAT antibody. New Figure S2 has images all using anti-STAT antibody for nos>me31B-RNAi and bam>me31B-RNAi (please note that the order of figure panels was changed to follow the new flow of the text, which was suggested by reviewers).

For the explanation on original Figure S2D (currently S2B), please see above (response to point 2).

4. If comparing RNA in situ levels and saying that loss of Me31B does not affect mRNA levels of nos, there should be quantification showing that there is no difference in RNA levels. In F and F' there appears to be higher levels of RNA in one of the wild type cysts when compared to the clone. Additionally, the size of the scale bars in Figure 3E-F are not stated.

We understand this reviewer's concern. We admit that it is quite tricky to provide the 'representative' images for these, because of multiple confounding factors: for quantification, we will need to capture the full depth of the cell/clone to integrate the signal intensity. But if we do so to generate the image, typically, it ends up including other cells that are present above/below the cyst of interest (in z-axis). As a result, it is almost impossible to capture representative images that is 'perfect' (where all SG cysts 'look' to have the same amount of nos mRNA, as actually is).

For this reason, we decided to provide quantification using the testis where me31B-RNAi is expressed in all germ cells. This is less aesthetic as you cannot have side-by-side comparison between control vs. RNAi cells, but the single molecule FISH allows us to compare the signal intensity (as 'number of signals') across different tissues. We now replaced the original images with new ones and added the quantification graph. Using this method, we did not detect difference in the nos mRNA level upon me31B-RNAi.

Also thank you for noticing the omission about the scale bars. It is 10μm, and we added this info to the legend.

5. Figure 3G lacks error bars. Additionally, according to the methods this IP experiment was performed from a NosGAL4>UAS-dpp background to enrich for SCs. This should be stated in the text to enhance reader clarity and prior literature cited to support this approach. Further, the observation that me31B binds to bam RNA should be contextualized as likely occurring prior to Bam

© 2021. Published by The Company of Biologists under the terms of the Creative Commons Attribution License (https://creativecommons.org/licenses/by/4.0/).

protein expression and differentiation. Finally, the authors should address the confounding nature of these results. Me31b is canonically a translational repressor, yet the authors show that loss of Me31B results in a delay in bam expression, despite the direct association between bam RNA and Me31b.

We modified the text to clearly state the use of Dpp in the main text.
Error bars for the technical replicates in Figure 3G are so small that it is not visible. We added the actual values to the figure legend.

6. The authors report that me31B knockdown leads to delayed Bam expression (Figure 3S). However, the authors use a version of Bam-GFP that has a functional bam promoter controlling expression of GFP-tagged Bam, but lacks the 5'UTR (at least according to the stock number listed). This version of Bam-GFP has been reported to express in GSCs in ovaries, however, this is not apparent from this study. It is unclear to this reviewer what this experiment is meant to demonstrate using this line.

We apologize for this mistake, and thank you for catching it. We obtained the Bam-GFP fly stock originally from Dr. Dennis McKearin, which we knew is a reporter that recapitulates endogenous bam expression. As you can see in Figure S3, indeed this reporter is expressed only after 4 SG stage (but not in GSCs). By checking back the notes, we're sure that the transgene we used is the one with endogenous bam UTRs and we corrected the reagents table.

7. The authors report that simultaneous upregulation of nos and me31B knockdown results in >32 cell SGs. This seems consistent with a differentiation defect rather than what would be expected by the authors proposed model which would be an excess of undifferentiated cells or dedifferentiating cells.

This figure is not meant to provide additional support to make the point of me31B being a regulator of dedifferentiation. We believe that (after the clarification and revision on above points raised by this reviewer) the role of me31B in dedifferentiation is already abundantly supported. Instead, the results described in this figure demonstrates synergistic effect of me31B knockdown and nos overexpression, which is much more drastic than me31B-RNAi only or nos overexpression only. We believe that the title (i.e. main conclusion) of this manuscript (me31B regulating dedifferentiation) is supported without this figure, but we felt that this figure adds to the contents, by providing drastic synergy between me31B-RNAi and nos overexpression, which we believe strengthens the role of me31B and nos in regulation of differentiation and dedifferentiation.

On a related note, we do see a lot of dedifferentiation-like figure in this condition (me31B-RNA + nos overexpression), including occurrence of cysts that contain more than 16 cells, but their fusome is not entirely connected. The phenotype is not a simple SG overproliferation as seen in dpp overexpression, as this reviewer might have assumed. We agree that we did not elaborate on this, thus we added some description of this phenotype in the main text ('The differentiation block was so severe that our criteria of dedifferentiation used above (i.e. connected cells at the hub with fragmented fusomes) was not applicable, although we frequently observed cysts with disintegrating fusomes, indicative of dedifferentiation' and some additional description based on rev#2's comments as well).

8. The authors report that simultaneous knockdown of me31B and nos greatly reduces the amount of germaria that contain dedifferentiating cysts. It would be helpful to the reader to see a representative image.

We have added the images as new Supplementary Figure S4. We originally felt that it would not add too much to the manuscript, as it would be just additional examples of testis apical tip with/without dedifferentiating cysts.

9. The scale bar is different in Figure 1E and E', size of scale bar should be stated in the legends.

Thank you for catching this. This is a photoshop mistake: the scale bar in E' must be a left over from past editing (not meant for the scale bar for E'). As E and E' are in the same magnification, no need for scale bar and thus removed. The bar in E is 10μm, which is now added to the legend.

© 2021. Published by The Company of Biologists under the terms of the Creative Commons Attribution License (https://creativecommons.org/licenses/by/4.0/).

10. The model in figure 6 suggests that me31B directly activates bam expression. The model could be misleading to a reader by showing an arrow as it may indicate that Me31B directly activates Bam translation. Additionally, the model represents both nos transcription and translation, however, the data presented do not differentiate between the two.

We appreciate this reviewer's comment on our model figure. We understand our model figure reflects ambiguity left surrounding the role of *me31B*, which needs to be clarified in the future studies. However, we believe that no model figure is expected to have every single point proven, or every single rationale elaborated. Not to mislead the readers, we have added a sentence in the figure legend that reads: 'Interactions between *me31B* and its targets indicated by arrows may be direct or indirect.'

However, please note that some of the concerns this reviewer expressed can be answered using logical reasoning and synthesizing from the data available in this manuscript, which we chose not to elaborate, because lengthy explanations will not help the readers' understanding.
➔ this reviewer states that 'the data presented do not differentiate between (the role of me31B in) nos transcription vs. translation'. However, we can fairly certainly say that it is most likely at the level of translation, at least for the matters concerning dedifferentiation. First, me31B's role in regulating dedifferentiation reside in ≥4 SG, because bam>me31B-RNAi results in dedifferentiation (Figure 1). In parallel, nos transcription reporter presented in Figure 6 suggests that nos transcription ceases after GB. Therefore, by the time cells reaches at 4-SG stage, where me31B functions to prevent dedifferentiation, nos transcription had ceased already for some time (2 cell cycle = about 24-32 hours). Therefore, it is difficult to imagine that <u>me31B represses nos transcription in 4 cell SG, after nos expression already had been turned off at the end of GB stage, to prevent dedifferentiation</u>, even if such a possibility cannot be entirely excluded. As a 'model' figure, we do not see the benefit of elaborating such a possibility only to disfavor after all.

11. In line 321-323 it states, "Overall, we conclude that me31B prevents dedifferentiation of SGs by reducing Nos protein levels and increasing Bam protein levels." However, the authors have not demonstrated that me31B directly increases bam protein levels.

We agree. We did not intend this to be the major conclusion (but we now see that it must have read that way). We added 'potentially' to read 'and potentially increasing Bam protein levels'.

Reviewer 2 Advance Summary and Potential Significance to Field:
The paper by Jensen et al builds a compelling case that Me31B-mediated translational repression of Nos regulates de-differentiation of spermatogonia in the Drosophila testis, and is thus critical to establishing and maintaining stem cell homeostasis. They also demonstrate that Me31B knockdown leads to hyperactivation of BMP signaling in germ cells, and that de-repression of Nos is sufficient for spermatogonial de-differentiation. Exactly how Me31B and Nos may regulate BMP signaling remains unclear. Nevertheless, the paper concludes with an interesting model, that 4- and 8-cell spermatogonia are delicately balanced as to whether to further differentiate or to de-differentiate, because they no longer transcribe nos RNA but are still able to translate it. In summary, I believe this manuscript makes a substantial and highly significant contribution to our understanding of molecular mechanisms underlying stem cell homeostasis.

Reviewer 2 Comments for the Author:
I believe the paper only requires minor revisions prior to publication. Most substantively, I would like to have more information about the striking differentiation block that occurs upon knockdown of me31B and overexpression of nos. In particular, what happens to the cysts that have undergone one or more additional mitotic divisions? Do they try to differentiate into spermatocytes and express markers for later stages of development, or do they just undergo cell death shortly after the developmental stage shown in Figure 5C? While not required in my view for this paper, further analysis of gene expression in these testes may identify additional Nos and Me31B targets with important roles in later cell differentiation events.

We thank this reviewer for the positive comments.

© 2021. Published by The Company of Biologists under the terms of the Creative Commons Attribution License (https://creativecommons.org/licenses/by/4.0/).

-Regarding the results described in Figure 5: the ‘complete block’ of differentiation by combined knockdown of me31B and overexpression of nos. To us, the most likely interpretation of this phenotype is that the SGs do not enter spermatocyte (SC) growth at all (in most cases). As a comparison, if SG tumor is induced by overexpression of Dpp, the testes exhibit the large expansion of SG, but the expanded SG region is followed by SC region that contains many cysts with a ‘size gradient’, suggesting that there is a continuous supply of new SCs (i.e. SGs committing to differentiation constantly, even though SGs overproliferate). In contrast, in me31B-RNAi + nos-overexpression, we do not see such ‘gradient in cell size’: even when SCs are present, they are very large without any smaller SCs between SGs and large SCs, suggesting that there has not been any new supply of SCs in the last several days (SC growth takes ~4 days). Those large SCs are likely from earlier developmental stages, before me31B-RNAi took effect.

It is likely that some overproliferated SGs die, but it is hard to quantify how frequently they die (because of the difficulty in setting an appropriate ‘denominator’): even in wild type, spermatogonia are constantly dying (Yang and Yamashita 2015, Development, Lu and Yamashita, 2017, eLife). In me31B-RNAi + nos overexpression testis, skewed ratio of cells makes it difficult to normalize the cell death frequency to compare to the wild type. We added a bit of description in the main text, without extrapolating too much.

-Regarding the gene expression analysis of me31B: indeed, this is clearly our next step, and we’re in the middle of 1) me31B-RIP-seq, and 2) RNA seq in me31B-RNAi. Unfortunately, we run out of time for the graduation of the first author, and we decided to leave those analysis for future studies.

I also have some small presentational comments.

Lines 36 and 39: Two consecutive sentences should not start with 'However...'; this is confusing.

The first ‘however’ was replaced by ‘in addition’, which still makes sense in the flow.

Line 248: 'of' is missing between 'deregulation' and 'STAT'.

Thank you for catching this. ‘of’ was added.

Line 255: 'of' is missing between 'activation' and 'STAT'.

Thank you for catching this. ‘of’ was added.

Lines 253-257: I think this material is more suitable for the discussion than for the results. The authors should also consider a different balance between the presentation of the somewhat inconclusive results with STAT, that are in a supplemental figure, and the clear results with BMP, that are in a main figure. I think it would be better to present the BMP results first and then the STAT results more briefly.

We agree. We switched the order of the presentation of BMP and STAT, and made the STAT description brief, and we moved some of discussion into the Discussion section.

Line 292: should read 'testes' not 'testis'

Corrected.

Line 321: I think bam RNA should be described as a 'potential additional target' for Me31B as there is only the RIP-qPCR evidence that supports this. In vivo effects could be indirect through Nos.

We edited the text according to this suggestion.

Line 390: replace 'to' with 'do'.

We replaced it with ‘and’ (which seemed to be more fitting upon careful reading).

© 2021. Published by The Company of Biologists under the terms of the Creative Commons Attribution License (https://creativecommons.org/licenses/by/4.0/).

Line 409: Insert 'in the' between 'bam' and 'germline'.

Thank you for catching this. We apologize that we had several typographical errors that we didn't catch prior to the initial submission. We went through the entire manuscript for additional rounds this time to catch typos.

Second decision letter

MS ID#: JOCES/2021/258757

MS TITLE: me31B regulates stem cell homeostasis by preventing excess dedifferentiation in the Drosophila male germline.

AUTHORS: Lindy Jensen, Zsolt G Venkei, George Watase, Bitarka Bisai, Scott Pletcher, Cheng-Yu Lee, and Yukiko M Yamashita
ARTICLE TYPE: Research Article

I am happy to tell you that your manuscript has been accepted for publication in Journal of Cell Science, pending standard ethics checks.
Reviewer 1

Advance summary and potential significance to field

Stem cell progeny close to self renewing signal still manage to differentiated efficiently. How these stem cell progeny differentiate efficiently instead of dedifferentiating is not fully understood. Jensen et al. describe a role for me31B in regulating male germline stem cell (GSC) differentiation by negatively regulating nos translation implicating translational control as a barrier to dedifferentiation.

Comments for the author

The authors have suitably addressed all of my concerns in the manuscript by adding data and by clarifying the text. Congrats to the all the authors on finishing this manuscript under these difficult circumstances.

Reviewer 2

Advance summary and potential significance to field

The paper by Jensen et al builds a compelling case that Me31B-mediated translational repression of Nos regulates de-differentiation of spermatogonia in the Drosophila testis, and is thus critical to establishing and maintaining stem cell homeostasis. They also demonstrate that Me31B knockdown leads to hyperactivation of BMP signaling in germ cells, and that de-repression of Nos is sufficient for spermatogonial de-differentiation.
Exactly how Me31B and Nos may regulate BMP signaling remains unclear. Nevertheless, the paper concludes with an interesting model, that 4- and 8-cell spermatogonia are delicately balanced as to whether to further differentiate or to de-differentiate, because they no longer transcribe nos RNA but are still able to translate it.
In summary, I believe this manuscript makes a substantial and highly significant contribution to our understanding of molecular mechanisms underlying stem cell homeostasis.

Comments for the author

My comments on the earlier submission have now been satisfactorily addressed.

© 2021. Published by The Company of Biologists under the terms of the Creative Commons Attribution License (https://creativecommons.org/licenses/by/4.0/).
